# Analysis of SARS-CoV-2 variants B.1.617: host tropism, proteolytic activation, cell–cell fusion, and neutralization sensitivity

**DOI:** 10.1080/22221751.2022.2054369

**Published:** 2022-04-07

**Authors:** Li Zhang, Qianqian Li, Jiajing Wu, Yuanling Yu, Yue Zhang, Jianhui Nie, Ziteng Liang, Zhimin Cui, Shuo Liu, Haixin Wang, Ruxia Ding, Fei Jiang, Tao Li, Lingling Nie, Qiong Lu, Jiayi Li, Lili Qin, Yinan Jiang, Yi Shi, Wenbo Xu, Weijin Huang, Youchun Wang

**Affiliations:** a Division of HIV/AIDS and Sex-transmitted Virus Vaccines, Institute for Biological Product Control, National Institutes for Food and Drug Control (NIFDC), Beijing, People's Republic of China; b Jiangsu Recbio Technology Co., Ltd., Taizhou, China; c Acro Biosystems, Inc., Beijing, People’s Republic of China; d CAS Key Laboratory of Pathogenic Microbiology and Immunology, Institute of Microbiology, Chinese Academy of Sciences, Beijing, People’s Republic of China; e Research Network of Immunity and Health (RNIH), Beijing Institutes of Life Science, Chinese Academy of Sciences, Beijing, People’s Republic of China; f National Institute for Viral Disease Control and Prevention, Chinese Center for Disease Control and Prevention, Beijing, People’s Republic of China

**Keywords:** SARS-CoV-2 variants, delta, B.1.617, infectivity, cell–cell fusion, neutralization

## Abstract

SARS-CoV-2 has caused the COVID-19 pandemic. B.1.617 variants (including Kappa and Delta) have been transmitted rapidly in India. The transmissibility, pathogenicity, and neutralization characteristics of these variants have received considerable interest. In this study, 22 pseudotyped viruses were constructed for B.1.617 variants and their corresponding single amino acid mutations. B.1.617 variants did not exhibit significant enhanced infectivity in human cells, but mutations T478K and E484Q in the receptor binding domain led to enhanced infectivity in mouse ACE2-overexpressing cells. Furin activities were slightly increased against B.1.617 variants and cell–cell fusion after infection of B.1.617 variants were enhanced. Furthermore, B.1.617 variants escaped neutralization by several mAbs, mainly because of mutations L452R, T478K, and E484Q in the receptor binding domain. The neutralization activities of sera from convalescent patients, inactivated vaccine-immunized volunteers, adenovirus vaccine-immunized volunteers, and SARS-CoV-2 immunized animals against pseudotyped B.1.617 variants were reduced by approximately twofold, compared with the D614G variant.

## Highlights

- B.1.617 variants exhibit enhanced infectivity in mouse ACE2-overexpression cells.
- B.1.617 variants caused increased cell–cell fusion.
- B.1.617 escaped from the neutralization of several mAbs.
- B.1.617 showed two-fold reduced neutralization sensitivity to vaccine elicited sera.

## Introduction

As of 8 Feb 2022, there were more than 394 million confirmed cases of COVID-19 worldwide, with a death total exceeding 5.7 million (https://covid19.who.int). Although several vaccines have been approved and numerous people have been vaccinated in many countries, the pandemic has not yet been effectively controlled. New local COVID-19 outbreaks are always accompanied by the emergence of new SARS-CoV-2 variants [1].

Since March 2021, there has been an outbreak of COVID-19 in India [2]. Viruses of the B.1.617 lineage have been identified as the main SARS-CoV-2 variants related to the outbreak of COVID-19 in India [2]. The B.1.617 variant was first discovered in India on October 2, 2020. Three sub-lineages B.1.617.1 (kappa), B.1.617.2 (Delta), and B.1.617.3 have been derived from the B.1.617 root lineage. By 8 Feb 2022, of the 7.99 million sequences in the GISAID database, 4.19 million are related to Delta. Mutation sites involved in the B.1.617 sub-lineages include T19R, T95I, G142D, E154K, F157del, R158del, L452R, T478K, E484Q, D614G, P681R, D950N, Q1071H, and H1101D. Three common mutations (L452R, D614G, and P681R) are shared by all viruses of the B.1.617 lineage. Additionally, two mutations exist in the receptor-binding domain (RBD) of the SARS-CoV-2 spike protein which are L452R and E484Q in B.1.617.1 and 3 or L452R and T478K in B.1.617.2 [3].

The major mutation sites of B.1.617 (e.g. L452R, E484Q, D614G, and P681R) are identical or similar to those in other globally circulating SARS-CoV-2 variants. Among them, L452R is the representative mutation site of variants B.1.427 and B.1.429 [4]. This mutation enhances binding with ACE2, increases viral infectivity, and reduces neutralization sensitivity [4,5]. The E484Q mutation site in B.1.617 is similar to mutations in variants B.1.351 and P.1(E484K), which is a key site that lead to immune escape [6–11]. The D614G mutation has spread fast and alters SARS-CoV-2 fitness, nearly all SARS-CoV-2 viruses thus far contain the D614G mutation [12–14]. Finally, the P681R mutation locates upstream of the furin restriction site (PRRAR) [15]. A similar mutation, P681H, has been identified in B.1.1.7, which were reported to promote cleavage of the S protein precursor [16] and affect O-glycosylation of the spike protein [17], but may not substantially impact viral entry or cell–cell spread [16].

In this study, we constructed a series of SARS-CoV-2 pseudotyped viruses based on the VSV system. We then systematically analysed the effects of B.1.617 variants on host tropism, protease hydrolysis, cell–cell fusion ability, and neutralization abilities of monoclonal antibodies, convalescent sera, and SARS-CoV-2 vaccine-immunized sera.

## Materials and methods

### Plasmids and pseudoviruses

The SARS-CoV-2 spike (GenBank: MN908947) protein expression gene was optimized using a mammalian codon and cloned into the pcDNA3.1 vector between BamHI and XhoI restriction sites. Site-directed mutagenesis was performed as described previously [18]; specific mutation sites and corresponding primers are presented in Supplementary Table. Fourteen ACE2-overexpression genes were optimized using a mammalian codon and cloned into the eukaryotic expression vector pRP[Exp]-EGFP-CMV between BamHI and XhoI restriction sites; the sources of these genes were human (BAB40370.1), mink (QNC68911.1), dog (MT663955), cat (MT663959), pangolin (XP_017505746.1), pig (NP_001116542.1), mouse (ABN80106.1), bat (KC881004.1), cow (NP_001019673.2), rabbit (MT663961), ferret (MT663957), sheep (XP_011961657.1), civet (AY881174.1), and monkey (MT663960) [19]. A FLAG tag (GACTACAAGGACGATGACGATAAG) was added at the 3’-terminal end of each gene. The dual split protein system (GFP_1-7_ RL_N_ / GFP_8-11_ RL_C_) was constructed as described by Kondo et al. [20]. Pseudotyped viruses of SARS-CoV-2 variants and single mutants were constructed in accordance with the methods described in our previous study.

### Cells

Five cell lines were used in this study: 293T (American Type Culture Collection, ATCC, CRL-3216), Vero (ATCC, CCL-81), LLC-MK2 (ATCC, CCL-7), Calu-3 (ATCC, HTB-55), and Huh-7 (Japanese Collection of Research Bioresources, Cat0403). The cell line 293T-hACE2 comprised 293T cells stably expressing human ACE2.

Fourteen ACE2 protein expression plasmids and two proteolytic enzyme transient overexpression cell lines were prepared by transfection of 293T cells using Lipofectamine 2000 (Invitrogen). All cells were cultured at 37°C in a 5% CO_2_ environment using Dulbecco’s modified Eagle medium (DMEM, high glucose; HyClone, Logan, UT) with 100 U/mL of penicillin–streptomycin solution (Gibco, Germany), 20 mM N-2-hydroxyethylpiperazine-N-2-ethane sulfonic acid (HEPES, Gibco) and 10% fetal bovine serum (FBS, Pansera ES, PAN-Biotech, Germany). Trypsin-EDTA (0.25%, Gibco) was used to detach cells for subculture at intervals of 2–3 days.

### Monoclonal antibodies

Sixteen anti-SARS-CoV-2 monoclonal antibodies (mAbs) were used in this study. The mAb sources were as follows: CB6 was from Dr. Jinghua Yan [21]; X593 and X604 were from Dr. X. Sunney Xie; SCTA01, H02M027, H014, H00S002 and HHV1 were from Dr. Liangzhi Xie of Sino Biological Company; 9G11, 4E5, and 7B8 were from Dr. Yuelei Shen of Beijing Biocytogen Inc.; AbG3 was from Dr. Zhiqiang He of Fapon Biotech Inc.; A261-262 was from Dr. Linqi Zhang of Tsinghua University; and A001, AM180, and AM128 were from Acro Biosystems Co.

### Convalescent sera

Serum samples from SARS-CoV-2 convalescent patients were provided by Dr. Wenbo Xu from the Chinese Center for Disease Control and Prevention. Nine samples were from patients in Beijing who had been infected with the D614G reference strain. They had been diagnosed with COVID-19 during the period from December 2020 to January 2021; the sera had been collected 14–28 days after discharge. Ten serum samples were from patients in Beijing who had been infected with the B.1.1.7 variant. They had been diagnosed with COVID-19 in January 2021; the sera had been collected 14–28 days after discharge. Written informed consent was obtained from all patients prior to blood collection. The study protocol involving convalescent serum samples was approved by the Ethic Committee of Chinese Center for Disease Control and Prevention

### Sera from vaccinated participants

Serum samples were collected from individuals who had been immunized with the inactivated vaccine [22] (KCONVAC, Shenzhen Kangtai Biological Products Co.; Chinese Clinical Trial Registry: ChiCTR2000038804); samples were collected at 14 days after the completion of a standard immunization procedure (doses at 0, 28, and 58 days; 5 µg/dose). Twenty samples were used in this study. Written informed consent was obtained from all volunteers prior to blood collection. The study protocol involving the inactivated vaccine was approved by the Ethic Committee of Jiangsu Provincial Center for Disease Control and Prevention.

Serum samples were collected from individuals who had been immunized with the adenovirus vaccine [23] (Ad5-nCoV, CanSino Biologics Inc. ChiCTR2000031781); samples were collected at 28 days after the completion of a standard immunization procedure (one dose at 0 days; 300 µl/dose). Eighteen samples were used in this study. Written informed consent was obtained from all volunteers prior to blood collection. The study protocol involving the adenovirus vaccine was approved by the Ethics Committee of Jiangsu Provincial Center for Disease Control and Prevention.

### Sera from immunized animals

Animals were handled under institutional (NIFDC, Beijing, China) guidelines for laboratory animal care and use, and the Animal Care and Use Committee at the NIFDC approved the animal study protocol.

Mice were immunized with purified SARS-CoV-2 plasmid comprising the D614G reference strain, B.1.351 variant, or B.1.429 variant (50 µg per mouse) at day 0. Pseudotyped viruses of the same SARS-CoV-2 variant in combination with aluminum adjuvant were used for the second and third immunization at days 14 and 28 respectively (6×10^5^ TCID_50_ per mouse). Blood samples were collected at 14 days after the third immunization. Serum samples from 10 mice were pooled (two mice per sample).

Horses were immunized using the SARS-CoV-2 RBD protein (original strain WH-1; RBD identical to the D614G reference strain) with Freund's incomplete adjuvant at an initial dose of 3 mg. Ten days later, they were immunized again using 6 mg of RBD protein with Freund's incomplete adjuvant. A third immunization was performed at 10 days after the second immunization, using 12 mg of RBD protein with Freund's incomplete adjuvant. Serum samples from four horses were collected at 7 days after the third immunization.

### Infectivity assay

Pseudotyped SARS-CoV-2 variants were serially diluted and mixed with Huh-7 cells or other indicated cells, then incubated at 37°C with 5% CO_2_. Twenty-four hours later, chemiluminescence signals were collected by the PerkinElmer Ensight device using Britelite plus reporter gene assay system (PerkinElmer) and displayed as relative luminescence units (RLUs). The detailed methods were described in our previous article [5]. Duplicate wells were established for each group. Each experiment was repeated four times. ACE2 expression levels were verified by FACS (Fig. S5).

### Neutralization assay

mAbs and serum samples were pre-diluted to specific initial concentrations. Serially diluted samples were then added to 96-well plates, mixed with pseudotyped virus, and incubated at 37°C for 1 h. Thereafter, 2 × 10^4^ Huh-7 cells/100 μL were added to each well of the 96-well plate. Cells were then incubated at 37°C with 5% CO_2_. Chemiluminescence signals were detected after 24 h. The ID_50_ (50% inhibitory dilution) was calculated using the Reed–Muench method. The results were recorded as the mean of three replicates.

### Proteolytic cleavage analysis

For each SARS-CoV-2 variant, 7 mL of pseudotyped virus were added to 2 mL of 25% sucrose buffer and centrifuged at 10,0000 g for 3 h. Each pellet of purified pseudotyped virus was then re-suspended in 100 μL PBS. Samples were mixed with loading buffer and heated at 100°C for 5 min; a 30-μL aliquot of each sample was then used for SDS-PAGE and western blotting analysis. The primary antibodies were a homemade mouse anti-S2 antibody against SARS-CoV-2 spike protein and a custom anti-VSV M (KeraFast, EB0011) protein antibody; the secondary antibody was a 1:10000 dilution of HRP-conjugated goat anti-mouse IgG (CWbiotech). Immobilon western chemiluminescent HRP substrate (Millipore) was used to develop the immunoreactive bands. Band intensities were calculated using Alphaview software.

### Cell–cell fusion assay

Donor cells were 293T cells that had been transfected with separate plasmids harbouring the spike genes of distinct SARS-CoV-2 variants or single mutants and the GFP_1-7_ RL_N_ plasmid. Acceptor 293T cells stably expressing human ACE2 were transfected with the GFP_8-11_ RL_C_ plasmid. The cells were incubated at 37°C with 5% CO_2_ for 24 h, then detached with trypsin. Spike protein expression levels were verified by FACS (Fig. S6). The donor and acceptor cells were mixed at a 1:1 ratio and seeded in 96-well plates. GFP and Renilla luciferase fluorescence values were monitored at 1–8 h after mixing. The GFP signals were collected using BioTek Cytation 5V. The EnduRen live cell substrate (Promega, E6481, WI) and Ensight device (PerkinElmer) were used for luciferase activity detection.

### Structural modelling

The spike protein was modelled based on the following Protein Data Bank coordinate sets: 7chh for X593, RBD-7B8 for 7B8, RBD-Ab5 for 9G11, and 7c01 for CB6; these revealed mutations L452R, T478K, and E484Q, respectively. PyMOL software (PyMOL Molecular Graphics System, Version 2.2.0, Schrödinger, LLC.) was used for visualization.

### Statistical analysis

GraphPad Prism 8 was used for plotting. One-way ANOVA and Holm–Sidak multiple comparisons tests were used for statistical analysis. Values are shown as means ± SEMs. Significance thresholds were as follows: *P < 0.05, **P < 0.01, ***P < 0.005, and ****P < 0.001.

## Results

### Sequence analysis of B.1.617 variants

**Figure 1.**
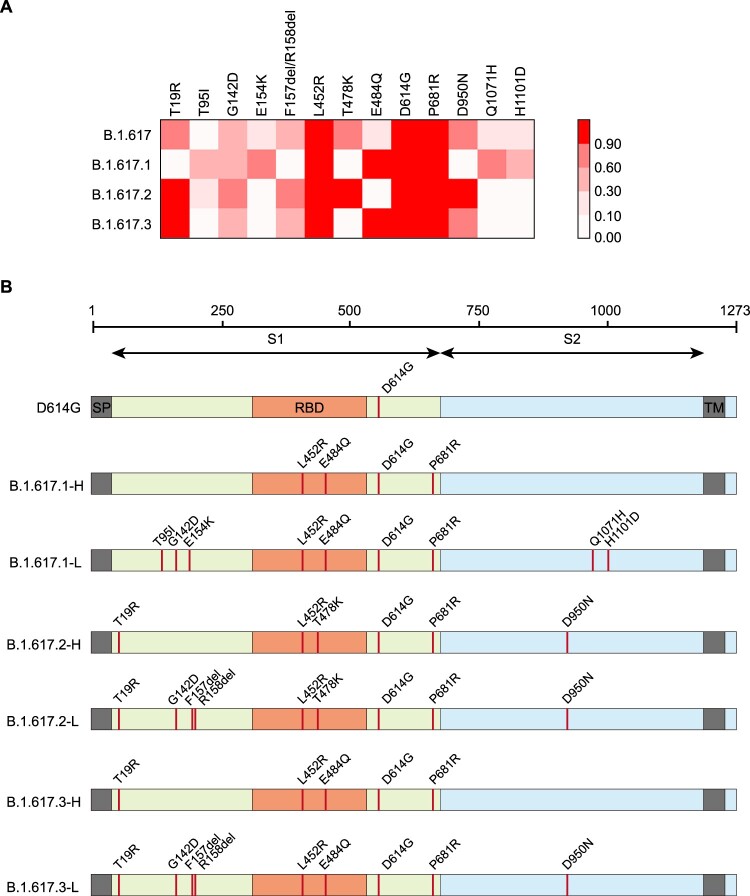
Analysis of mutations in B.1.617 variants. A. Mutation sites with frequencies of >30% in at least one B.1.617 sub-lineage were tracked using an outbreak website. The heatmap shows the proportions of sequences with each mutation among all sub-lineage sequences. B. Diagrammatic sketch of B.1.617 variants that were constructed as pseudotyped viruses and analysed in this study.

According to data from the Outbreak.info project, 758 906 sequences were classified as belonging to B.1.617 lineages as of Feb 8, 2022. Among them, 7792, 150 780 and 328 sequences respectively belonged to the B.1.617.1, B.1.617.2 and B1.617.3 sub-lineages, while the remaining unclassified 6 belonged to the B.1.617 root lineage [3]. The proportions of distinct single mutations were compared among lineages. Based on mutation frequency differences, each of three B.1.617 sub-lineages was defined as H for high-frequency variants (comprising mutations with >90% frequency) and L for low-frequency variants (comprising mutations between 30% and 90% frequency) (Figure 1(A)). Pseudotyped viruses for all the three sub-lineages with both high- and low-frequent mutations were constructed. Meanwhile, all single point mutations composed the variants, as well as pairwise combinations of mutations in the RBD region, was also constructed. The D614G single point mutation based on the original strain was used as the reference sequence (Figure 1(B)).

### Infectivity and animal tropism

**Figure 2.**
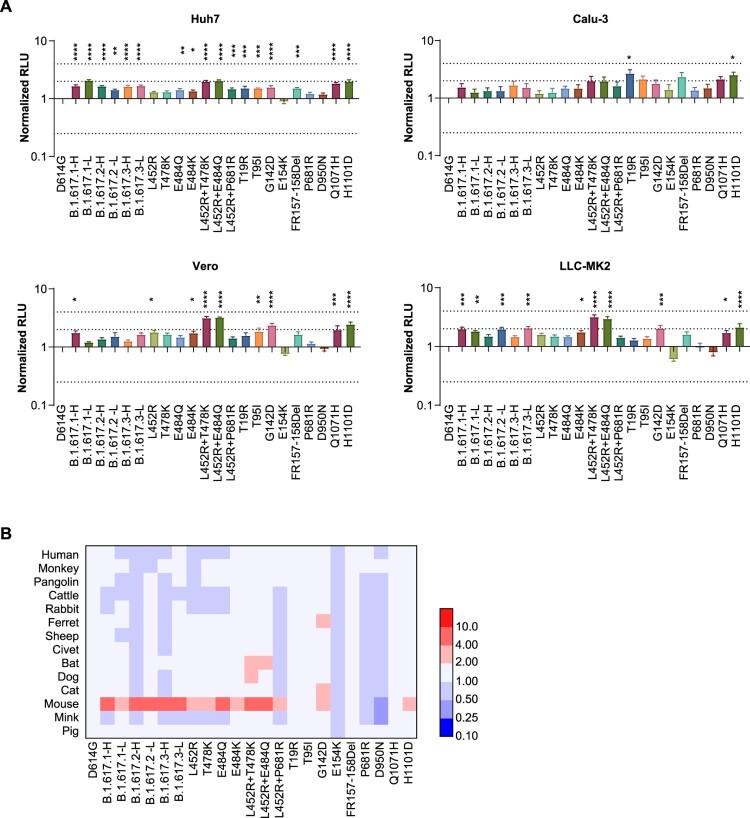
Analysis of B.1.617 infectivity. A. Normalized chemiluminescence signals (in RLUs) of target cells were calculated compared with the D614G reference strain. Data represent the results of four replicate experiments. Dotted lines indicate twofold and fourfold change. B. Equal amounts of ACE2-overexpression plasmids from different species were transfected into 293T cells. Ratios of infectivity compared with the D614G reference strain were shown. Data representing the results of four replicate experiments are shown in heatmap format. Red represents increased infectivity and blue represents decreased infectivity.

The infectivities of B.1.617 variants were slightly increased in four SARS-CoV-2 susceptible cell lines: Huh-7, Vero, Calu-3, and LLC-MK2 (less than twofold). Investigation of single mutations indicated that the Q1071H and H1101D single mutations and the combinations of L452R with T478K or E484Q could slightly enhance infectivity (Figure 2(A)).

To investigate the host range changes of B.1.617 variants, we tested 14 ACE2-overexpressing 293T cell lines from various species. The results suggested that the RBD-specific mutations L452R, T478K, and E484Q significantly enhanced viral infectivity of mouse ACE2-overexpressing cells, compared with the D614G reference strain. Although the viral infectivity for other species did not change over 4-fold among B.1.617 sub-lineages, the L452R+T478K, L452R+E484Q, T95I, G142D, Q1071H, and H1101D mutations led to increased infectivity in most species (Figures 2(B) and S1). In addition, the infectivity of the Delta variant (B.1.617.2) was not changed or was slightly decreased in most of the investigated species except mice. Interestingly, the infectivity of B.1.617.1 was slightly increased in cells overexpressing ACE2 orthologs from bats, ferrets, dogs and mink (Figures 2(B) and S1).

### Proteolytic enzyme effects

**Figure 3.**
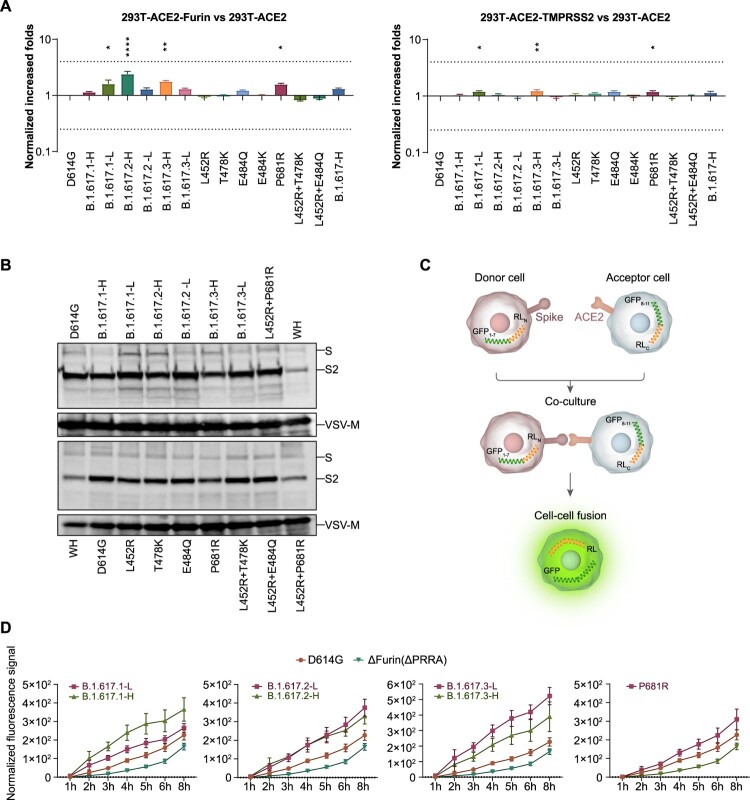
Analysis of proteolytic activity and cell–cell fusion. A. Proteolytic enzymes furin and TMPRSS2 were separately overexpressed in 293T-hACE2 cells. Data shown indicate relative infectivity changes because of enzyme overexpression. Relative RLUs were compared with or without the indicated enzyme first, then compared with the D614G reference strain. Results were obtained from four independent experiments. Dashed lines indicate the threshold of fourfold difference. B. B.1.617 and reference pseudotyped viruses were centrifuged in sucrose buffer, then resuspended in PBS for SDS-PAGE. Western blotting was performed with mouse anti-S2 polyclonal antibodies. VSV-M was used as an internal control. Representative results of three replicate experiments are shown. C. Diagrammatic sketch of dual reporter cell–cell fusion system. 293T cells were used as donor cells. D. Time course curve of cell–cell fusion. Fluorescence signals of GFP were normalized to the signal of the D614G reference strain after 1 h of co-incubation; values shown indicate means ± SEMs. Representative results of three independent experiments are shown.

Because all B.1.617 variants carry the P681R mutation, adjacent to the proteolytic site, we investigated the influence of protease overexpression (using multiple proteases) on viral infectivity. The increased infectivities by furin overexpression in B.1.617 variants were slightly greater than that of the D614G reference strain (Figure 3(A)). A similar phenomenon was not observed upon TMPRSS2 overexpression.

We subsequently investigated enzyme proteolytic activities by examining the proteolysis of S1 and S2 proteins in pseudotyped virus particles. As shown in Figure 3(B), the S2 proportion was not significantly increased in B.1.617 variants, RBD single mutants, or P681R mutants, compared with the D614G reference strain.

### Effects on cell–cell fusion

To examine whether the S proteins of B.1.617 variants and the P681R mutant influenced cell–cell fusion characteristics, a dual reporter system consisting of a pair of split Renilla luciferase (spRL) fused to split green fluorescent protein (spGFP) was used [20]. The strengths of the luciferase or GFP signals indicated the degree of host cell fusion (Figure 3(C)). The luciferase and fluorescence signals were monitored from 1 to 8 h after donor and acceptor cells had been mixed. Pseudotyped virus without the classical furin (delta PRRA) site was used as a negative control. The fluorescence signals were 1.2–2.3-fold higher in B.1.617 variant-infected cells than in D614G reference strain (Figure 3(D)). The luciferase signals were also compared (Fig. S2), the changes were less obvious than fluorescence signal. Further analyses based on single mutations suggested that the P681R single mutation also enhanced cell–cell spread (Figure 3(D)).

### Monoclonal antibody neutralization

**Figure 4.**
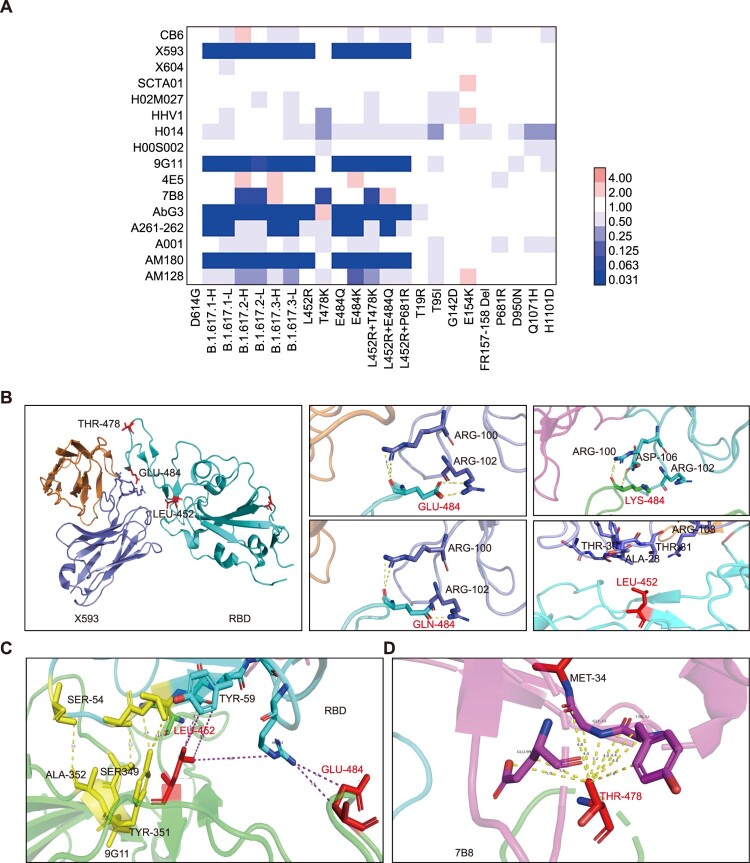
Neutralization activities and structural analyses of mAbs against B.1.617 variants and single point mutations. A. Data show the neutralization ID50 ratio of each variant, compared with the D614G reference strain. Red represents increased neutralization capacity and blue represents decreased neutralization capacity. B. Structural modelling of the L452R, T478K, and E484Q mutations, based on 7chh for X593, RBD-7B8 for 7B8, RBD-Ab5 for 9G11, and 7c01 for CB6.

Monoclonal antibodies (mAbs) against the SARS-CoV-2 spike protein offer promising therapies for COVID-19. We examined the neutralization effect of 16 mAbs, including one mAb in clinical use (CB6) [21] and 17 mAbs currently under investigation. The results suggested that B.1.617.1-H/L and B.1.617.3-H/L variants mainly escaped the X593, 9G11, AbG3, A261-262, and AM180 mAbs. These effects were presumably caused by a L452R and E484Q double mutation in the RBD region. These findings were verified by analyses of pseudotyped viruses with single and combined mutations of L452R and E484Q in the RBD region. B.1.617.2-H/L, which exhibited L452R and T478K mutations in the RBD region, reduced the neutralization effects of the X593, 9G11, 7B8, AbG3, and AM180 mAbs. Notably, although E484Q and E484K both involve an identical mutation site, E484K allowed escape from the AM128 mAb, whereas E484Q did not. We did not find any impacts of other single point mutations outside the RBD region on the neutralization effects of mAbs (Figure 4(A), see also Figs. S3 and S4).

### Structural analysis

We further analysed the structure of the spike and antibody complex, based on published data. Concerning the X593 mAb, E484 and L452 are located at the RBD–antibody binding interface. Mutation of E484Q creates a charge leads to disruption of the interaction, while mutation of L452R affects the hydrophobic interactions. The mAb 7B8 was the only antibody from which T478K could escape in this study. Mutation of T478K destroys the hydrophobic environment, thus affecting the RBD–antibody interaction. As for mAb 9G11, although L452 and E484 are near the interaction surface, they exhibit minimal interaction forces. The L452R mutation may produce a charge conflict with R64, resulting in reduced affinity. Additionally, the mutations L452R, T478K, and E484Q are far from the binding site with the mAb CB6; thus, they do not directly affect its neutralization interactions (Figure 4(B–D)).

### Convalescent sera neutralization

**Figure 5.**
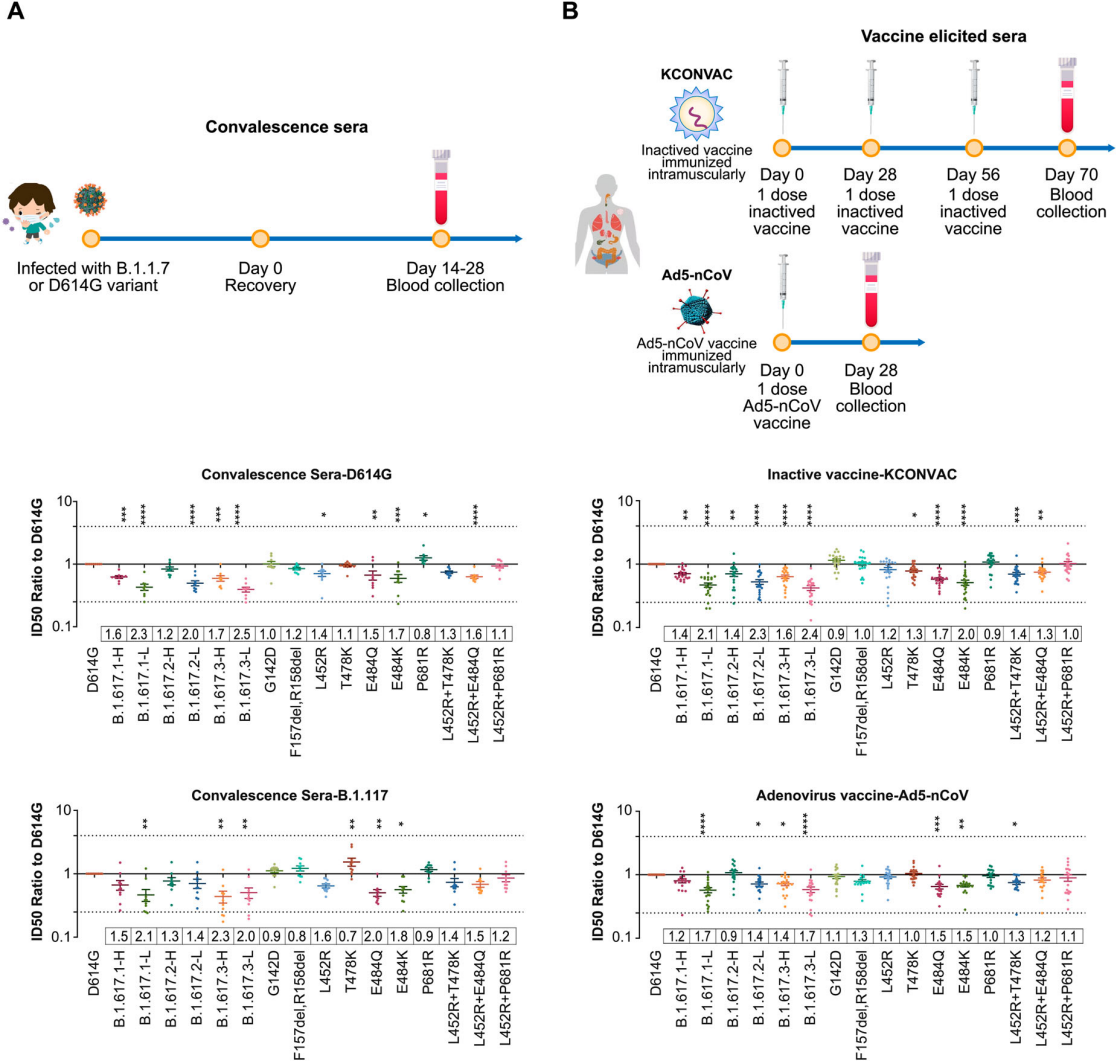
Neutralization activities of convalescent sera and vaccine elicited sera. Normalized ID50 ratios compared to D614G reference strain are shown. Means ± SEMs are shown for each variant. Dashed lines indicate the threshold of fourfold difference. Reduced differences (compared with the D614G reference strain) are labelled at the bottom of each plot. All experiments were repeated 2–4 times, depending on sample availability. A. Neutralization activities of convalescent sera. B. Neutralization activities of vaccine-immunized sera.

As for patients infected with D614G variant, the B.1.617.1-H/L and B.1.617.3-H/L variants reduced the neutralization activities of convalescent sera by 1.6–2.5-fold, while B.1.617.2-H/L variants reduced the neutralization activities of convalescent sera by 1.2–2.0-fold. Single RBD mutations analysis suggested that L452R or E484Q mutations (including L452R combined with E484Q or T478K mutations) were related to the reduced neutralization effect. Our mutation analysis results also suggested that the E484Q mutation had a greater effect on neutralization than the T478K mutation. Additionally, neutralization against B.1.617-L was more strongly reduced, compared with neutralization against B.1.617-H, suggesting that mutations outside the RBD also affect the neutralization activities of convalescent sera (Figure 5(A)).

We also analysed serum samples from convalescent patients who had been infected with the B.1.1.7 variant. Compared with D614G, the neutralization effects against B.1.617.1-H/L and B.1.617.3-H/L variants were reduced by 1.5–2.3-fold, while the neutralization effects against B.1.617.2-H/L variants were reduced by 1.3–1.4-fold (Figure 5(A)).

### Vaccine-immunized sera neutralization

The protective abilities of two vaccines that have been approved in China have been tested: an inactivated vaccine [22] and an adenovirus vector vaccine [23]. Compared with the D614G reference strain, the B.1.617.1-H/L, B.1.617.2-H/L, and B.1.617.3-H/L variants reduced the neutralization activities of inactivated vaccine-immunized sera by 1.4–2.1, 1.4–2.3, and 1.6–2.4-fold, respectively; and reduced the neutralization activities of adenovirus vaccine-immunized sera by 1.2–1.7, 0.9–1.4, and 1.4–1.7-fold, respectively. Notably, the low-frequency variants with more mutations consistently reduced the neutralization abilities to a greater extent, compared with the high-frequency variants. Single-mutation analyses indicated that E484Q was the main source of neutralization resistance, whereas the L452R and T478K mutations showed weaker effects. Furthermore, E484Q induced neutralization resistance to an extent comparable with the resistance induced by E484K. Overall, although the B.1.617 variants reduced the neutralization abilities of various vaccines by approximately 0.9–2.4-fold, suggesting both vaccines continued to exhibit a protective effect (Figure 5(B)).

### Neutralization of animals immunized sera

**Figure 6.**
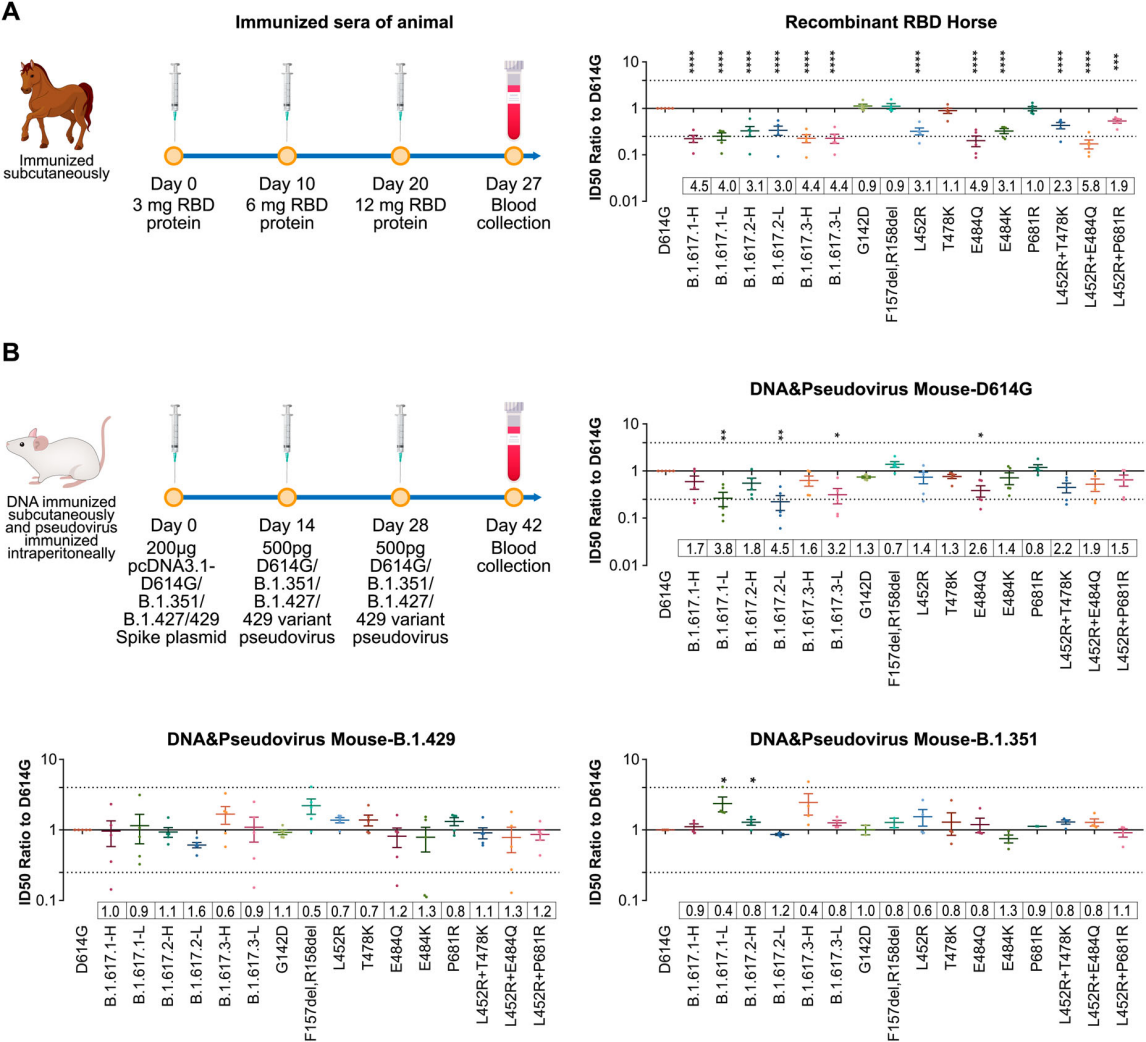
Neutralization activities of from animals immunized with D614G and other SARS-CoV-2 variants. Normalized ID50 ratios compared to D614G reference strain are shown. Means ± SEMs are shown for each variant. Dashed lines indicate the threshold of fourfold difference. Reduced differences (compared with the D614G reference strain) are labelled at the bottom of each plot. All experiments were repeated 2–4 times, depending on sample availability. A. Neutralization activities of RBD protein-immunized horse sera. B. Neutralization activities of sera from full-length spike DNA-immunized and pseudotyped virus-immunized mice. Immunization procedures are shown in the left panel.

We first tested neutralization activities of RBD protein-immunized horse sera. Their neutralization activities were significantly reduced (by 3-4-fold) against all B.1.617 variants, as well as the L452R, T478K, and E484Q single or double mutants. (Figure 6(A)). The neutralization activities of sera from animals immunized with other variants (e.g. D614G, B.1.351, and B.1.429) were also tested. Full-length spike DNA plus pseudotyped virus was used to immunize mice, yielding a series of post-immunization sera. Analysis of immunized sera suggested that antisera obtained by immunization with B.1.351 and B.1.429 immunogens showed no decreased neutralization activities against B.1.617 variants, compared with the D614G reference strain (Figure 6(B)).

### Discussion

In India, the prevalence of B.1.617 sub-lineages has increased in a manner consistent with the surge of COVID-19 cases [2,3]; Among them, B.1.617.1, especially B.1.617.2 comprise most of sequences [3]. Furthermore, the proportion of B.1.617.2 sequences is markedly increasing, whereas the proportion of B.1.617.3 sequences is limited [3]. The B.1.617.2 variant was identified as the fourth VOC Delta by WHO at May 2021, and became the predominant variant worldwide during the second half of the year 2021 [24]. Although Omicron is increasing fast since Dec 2021, Delta is still transmitted in some area, and the SARS-CoV-2 variant with the highest pathogenicity so far [25] (GISAID).

The B.1.617.1 and B.1.617.2 variants are predicted to have increased transmissibility [26]. Previous studies concern SARS-CoV-2 mutations [27] and a structural analysis of B.1.617 major mutations (L452R, E484Q, and P681R) suggested increased ACE2 binding by these variants [28]. Our results suggested entry abilities into Huh-7, Calu-3, Vero, and LLC-MK2 cell lines were not significantly increased. Notably, similar as K417N, N501Y, and E484K in the B.1.351 variant [8], RBD mutations in the B.1.617 variants also showed enhanced infectivity in mouse cells. Additionally, RBD mutations in the B.1.617 variants also led to enhanced infectivity in mouse cells. Additionally, the mouse-adapted mutants N501Y, Q493K, and Q498H of the SARS-CoV-2 RBD also showed significantly increased binding affinity towards mouse ACE2 [29,30]. Therefore, mice must be closely monitored as a potential host of SARS-CoV-2. The causes of increased affinity for mouse ACE2, as well as the structural differences between mouse and human ACE2 proteins, require further analyses. The SARS-CoV-2 spike protein is hydrolyzed to S1 and S2, which is the first step of virus infection and the prerequisite for virus–cell fusion [31,32]. Furin, TMPRSS2, and cathepsin L are key proteases that mediate the hydrolysis of SARS-CoV-226. SARS-CoV-2 exhibits much greater capacity for membrane fusion than does SARS-CoV because of the PRRA (681–684) insertion into the S1 and S2 junction [33]. B.1.617 variants contain a P681R mutation, which is located adjacent to this cleavage site; structural prediction has suggested that the S1–S2 clearance rate of B.1.617 might be affected. Our results suggested that the B.1.617 variants are slightly more sensitive to furin overexpression.

Cell–cell fusion provides an additional route for viral dissemination throughout the host. Because the ability to transmit between cells is particle-independent, immune factors (e.g. antibodies) have been presumed to poorly block this type of spread [34]. The development of specific inhibitors targeting the cell–cell fusion process is also a potential antiviral strategy [34]. By using the dual split reporter system, we found a twofold greater tendency for fusion in B.1.617 variants, compared with the D614G reference strain. These results suggest that the increased furin activity and cell–cell spread may explain the increased transmissibility and pathogenicity of B.1.617. In addition, several related studies were consistent with our results. Saito et al. reported that P681R enhances viral fusion, and the P681R mutant virus exhibited higher pathogenicity compared with its parental strain in infected hamsters [35]. Similarly, Zhang et al. also suggested that Delta spike protein can fuse membranes more efficiently and infect cells faster than other SARS-CoV-2 variants [36].

E484K-mediated immune escape has been reported by many groups [6]; this comprises a key mutation in many VOCs and VOIs (e.g. B.1.351, P.1, P.2, B.1.525, and B.1.526) that can escape from both mAbs and vaccine-immunized sera [1]. Mutations at site 484 have also been identified in the context of therapeutic mAb selective pressure and during persistent infection in immunocompromised hosts [37,38]. The L452R mutation has also been discovered in some VOIs, such as B.1.427 and B.1.429; it reportedly reduces or abolishes the neutralization activities of several mAbs, allowing escape from vaccine-immunized sera [39]. The B.1.617 variant contains both E484 and L452 mutations, which presumably alter viral antigenicity. A recent study showed that the B.1.617.1 variant was resistant to the mAb bamlanivimab, but it was not resistant to imdevimab or a cocktail of casirivimab and imdevimab [40]. In our study, we tested 16 mAbs; the effects of four were reduced by all B.1.617 sub-lineages, whereas the effects of two were abolished by either B.1.617.1 and B.1.617.3 or B.1.617.2 variants because of differences involving T478K and E484Q mutations. Additionally, we compared the E484Q and E484K mutations. While the 9G11, AbG3, and AM128 mAbs were affected more by the E484K mutation, the X593 and AM180 mAbs reacted similarly to viruses containing either mutation. Notably, only one antibody (A261-262) exhibited distinct reactions to L452R and E484Q, which indicated that the two mutations may be located in nearby epitopes. We did not observe considerable synergistic effects between L452R and E484Q or T478K and E484Q. Structural analysis could explain these results: mutations in the B.1.617 variant are precisely at the mAb–spike interface. MAb cocktails are highly recommended in clinical treatment; specific mAbs are needed for particular SARS-CoV-2 variants.

Reduced neutralization activities against B.1.617 variants were reported for mRNA-1273 vaccine-elicited sera (3–7 fold) [41], and inactivated vaccine BBV152 (1.84 fold) [42]. We tested two vaccines approved in China: inactivated vaccine (KCONVAC) [22] and adenovirus vaccine (Ad5-nCoV) [23]. The neutralization activities of which reduced approximately twofold. In addition, RBD-immunized horse sera were tested to compare the effect of RBD or full-length spike. Our results showed more obvious reduction (3-4 fold) of RBD-elicited sera against B.1.617 than the inactivated or adenovirus vaccine-elicited sera which using full-length Spike protein as immunogen(2-fold). To predict whether the vaccine based on B.1.429 or B.1.351 variants or previous infection of these variants would provide protections against B.1.617, mice were immunized using pseudoviruses of B.1.429(containing L452R), B.1.351 (containing E484K) and D614G. The results revealed that the immune sera did not reduce neutralization activity against the B.1.617 variants, compared with the D614G reference strain, suggesting that key mutation sites (e.g. L452 and E484) shared in B.1.617 and B.1.429 or B.1.351 play an important role in immunogenicity.

When the neutralization sensitivities were compared among B.1.617 variants (B.1.617.1, B.1.617.2 and B.1.617.3), the neutralizing antibody titers of low-frequency B.1.617 variants with more mutation sites were always more obviously reduced, compared with the high-frequency B.1.617 variants. This indicated that non-RBD mutations may be responsible for the further reduction of neutralization sensitivity, which was in agreement reports about neutralizing mAbs against NTD [43] and the SARS-CoV-2 co-receptor AXL, which also binds to the NTD [44]. Furthermore, T19R and G142D mutants were found to escape from several NTD-specific mAbs by McCallum et al. [43].

The reduced neutralization activities against B.1.617 variants in vitro in accordance with the real-world data of increased infections [45]. However, the antibody-escaping capacity dose not equal to the increased breakthrough infection. Another reason may be the fast waning of vaccine or infection elicited neutralization antibodies, as well as the barrier IgA protection [40]. Therefore, a booster dose maybe quite helpful, as it could enhanced the neutralization antibody to a large extend. Moreover, research by Khoury et al. showed that the level of antibody for neutralization for 50% protection against detectable SARS-CoV-2 infection was 20.2% of the mean convalescent antibody level. To protect against severe disease, 3% of the mean convalescent antibody level is sufficient [46]. Neutralization activities against B.1.617 were reduced by approximately twofold in vaccine-immunized sera, suggesting that the current vaccines are protective against severe disease caused by B.1.617.

In conclusion, our findings indicate that the host range of B.1.617 variants may not considerably differ from the D614G reference strain, although the mouse transmission characteristics showed notable differences. B.1.617 showed slightly promoted cell–cell fusion, which may influence its pathogenicity and transmissibility. Three RBD mutations in B.1.617 can cause immune escape from multiple mAbs, which should be carefully considered in clinical mAb therapy. In patients infected with the D614G reference strain or the B.1.1.7 variant, convalescent serum neutralization activities are decreased by approximately two-fold. Neutralization activities were reduced by only around two-fold in vaccine-immunized sera, suggesting that the current vaccines remain protective.
